# Morphometric assessment of tibial nerve and its branches around the ankle

**DOI:** 10.1097/MD.0000000000037745

**Published:** 2024-04-12

**Authors:** Jeha Kwon, Hong Bum Park, Soonwook Kwon, Im Joo Rhyu, Dong Hwee Kim

**Affiliations:** a Oxford University Hospitals NHS Foundation Trust, Oxford, United Kingdom; b Department of Physical Medicine and Rehabilitation, College of Medicine, Korea University, Ansan, Republic of Korea; c Tatoa Clinic, Seoul, Republic of Korea; d Department of Anatomy, Korea University College of Medicine, Seoul, Republic of Korea; e Division of Brain Korea 21 Plus Program for Biomedical Science, Korea University College of Medicine, Seoul, Republic of Korea.

**Keywords:** tarsal tunnel, tarsal tunnel syndrome, tibial nerve, tibial neuropathy

## Abstract

It is essential to understand the considerable variations in bifurcation patterns of the tibial nerve (TN) and its peripheral nerves at the level of the tarsal tunnel to prevent iatrogenic nerve injury during surgical nerve release or nerve block. A total of 16 ankles of 8 human cadavers were dissected to investigate the branching patterns of the TN, using 2 imaginary lines passing through the tip of the medial malleolus (MM) as reference lines. Bifurcation patterns and detailed information on the relative locations of the medial plantar, lateral plantar, medial calcaneal, and inferior calcaneal nerves to the reference lines were recorded. The most common bifurcation pattern was Type 1 in 12 ankles (75%), followed by Type 2 in 2 ankles (13%). One medial calcaneal nerve (MCN) was seen in 11 (69%) specimens and 2 MCN branches were seen in 5 (31%) specimen. 88% of the MCN branches bifurcated from the TN, whereas 6% originated from both TN and lateral plantar nerve (LPN). At the level of the tip of the MM, 2 of 7 parameters showed statistically significant difference between both sexes (*P* < .05). There was a statistically significant difference between left and right ankles in 2 of 7 measurements (*P* < .05). Further morphometric analysis of the width, distance, and angle between the TN branches and the tip of MM showed a highly variable nature of the location of the peripheral nerve branches.

## 1. Introduction

The tibial nerve (TN) originates from the L4-S3 spinal nerve roots and branches into the medial and lateral plantar nerves (LPNs) and the medial and inferior calcaneal nerves (ICNs). Compression of the TN and its branches in the tarsal tunnel (TT) resulting in tarsal tunnel syndrome (TTS) has been well-described however, it remains a relatively uncommon and underdiagnosed clinical condition.^[1–4]^

Although many textbooks report the variations of the branching patterns of the TN with emphasis on the medial and LPNs, variations of the calcaneal branches have not been widely described in current literature.^[5–8]^ It is essential to understand the possible TN branches around the ankle in order to better diagnose and treat heel pains caused by TTS. For example, as the medial calcaneal nerve (MCN) branch provides sensory innervation to the medial aspect of the heel and part of the calcaneus, its division proximal to the TT spares the heel in TTS.

Therefore, the aim was to conduct morphometric assessment of the TN branching patterns, to enable their accurate localization.

## 2. Methods

Sixteen ankles of 8 human cadavers (4 males and 4 females; median age, 70 years) were dissected to investigate the branching patterns of the TN. The exclusion criteria included cadavers with heel injury or trauma, ankle or distal tibia fracture, and foot amputation prior to dissection. Each foot was placed in its anatomically neutral position and the skin, fascia, and subcutaneous tissue were carefully dissected starting 20 cm proximal to the medial malleolus (MM) along the course of the TN to the plantar surface. The institutional review board confirmed that this cadaveric study was not subject to review in the absence of relevant laws when conducting the study, and the consent was waived.

**Figure 1.**
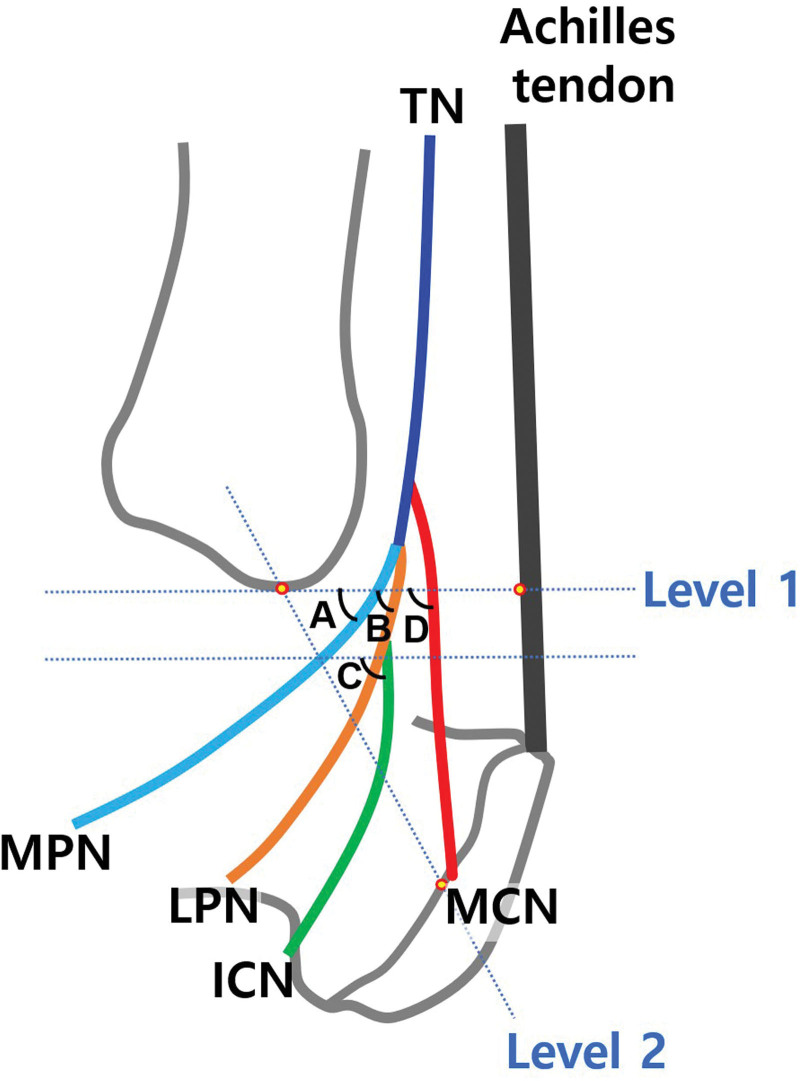
Anatomical landmarks and levels used for measurements of TN and its branches. Level 1 is the horizontal line at the tip of medial malleolus. Level 2 is the oblique line from the tip of medial malleolus and the midpoint of calcaneus. A = the angle between MPN and the horizontal line at Level 1, B = the angle between LPN and the horizontal line at Level 1, C = the angle between ICN and the horizontal line at its branching point, D = the angle between MCN and the horizontal line at the Level 1, ICN = inferior calcaneal nerve, LPN = lateral plantar nerve, MCN = medial calcaneal nerve, MPN = medial plantar nerve, TN = tibial nerve.

**Figure 2.**
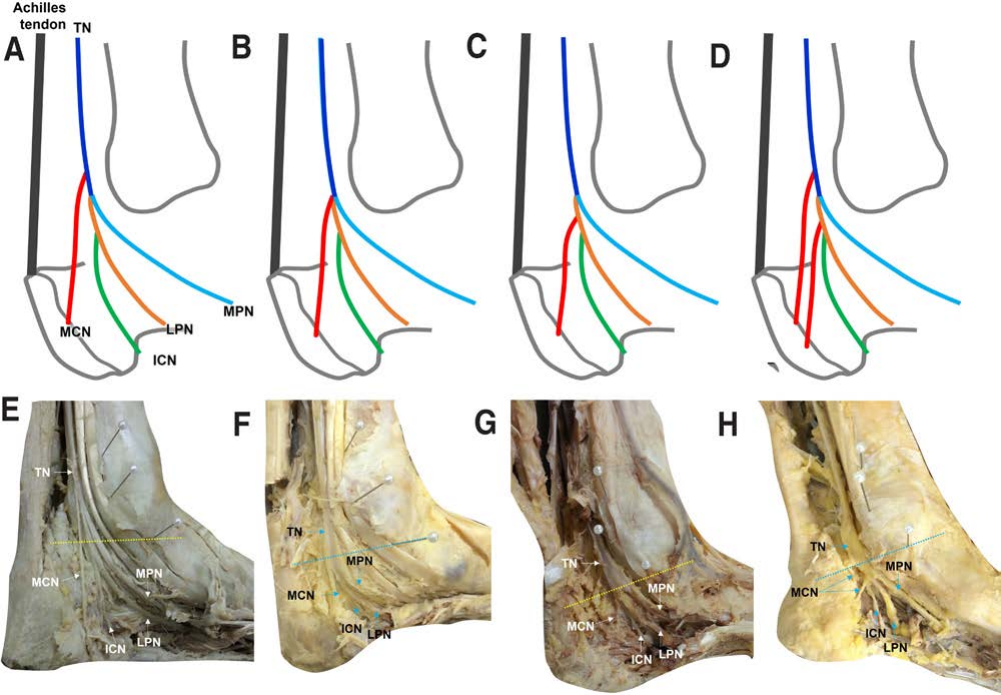
Patterns of TN and its branches. Type 1, MCN branching proximal to the bifurcation point of medial and lateral plantar nerves (A, E); Type 2, MCN branching at the bifurcation point of MPN and LPN from TN (B, F); Type 3, MCN branching from LPN (C, G); Type 4, MCN branching from TN and LPN (D, H). LPN = lateral plantar nerve, MCN = medial calcaneal nerve, MPN = medial plantar nerve, TN = tibial nerve.

The tip of the MM and the midpoint of the calcaneus were used to draw imaginary horizontal and oblique straight lines. Level 1 represented a horizontal line at the tip of the MM and level 2 represented an oblique line from the tip of the MM to the midpoint of the calcaneus (Fig. 1). The midpoint of the calcaneus was defined as the point half way between the Achilles tendon insertion and part of the calcaneus bone which makes contact with the floor. The distances between these reference lines and the TN bifurcation points were measured using a Vernier caliper. Positive and negative values indicated branching points proximal to and distal to the reference lines, respectively. The branching pattern of the MCN was categorized into 4 types (Type 1, MCN branching proximal to the bifurcation point of medial and LPNs; Type 2, MCN branching at the bifurcation point of medial plantar nerve [MPN] and LPN from TN; Type 3, MCN branching from LPN; Type 4, MCN branching from TN and LPN) (Fig. 2). This classification was adapted from a previous study.^[9]^ The angles between the TN branches and the horizontal line at Level 1 were measured using a protractor (A, B, D, Fig. 1). In cases were ICN branched distal to Level 1, the angle between ICN and the horizontal line at its branching point was measured (C, Fig. 1). Patterns of TN branches were also noted.

All statistical analyses were performed using Statistical Package for Social Sciences (version 20.0; SPSS Inc., Chicago, IL). Nonparametric tests were conducted owing to the small sample size and the non-normal distribution of some parameters for which the median with minimum and maximum values were provided. Parameter differences according to sex were analyzed using the Wilcoxon rank-sum test. Differences in measurements between the left and right sides of each cadaver were assessed using Wilcoxon signed-rank test. Statistical significance was considered at *P* < .05.

## 3. Results

Of the 16 specimens, 11 (69%) had a single MCN and 5 had 2 (31%) MCN branches. In 4 of 5 (80%) ankles where more than 1 MCN branch was seen, the bifurcation pattern was Type 1, and in 1 of 5 ankles (20%), the branching pattern was Type 4. Two MCN branches were seen in 3 (37.5%) male (n = 8) and 2 (25%) female (n = 8) specimens, and in 2 (25%) left (n = 8) and 3 (27.5%) right (n = 8) ankles. The origin of MCN branches was most commonly from the TN in 14 of 16 (88%) ankles; the proportion was 100% (8 ankles, n = 8) in left and 75% (6 ankles, n = 8) in right ankles. MCN branches also arose from the LPN and both TN and LPN in 1 (6%) ankle each. The most common bifurcation pattern was Type 1 in 12 cases (75%), followed by Type 2 in 2 cases (13%), Type 3 in 1 case (6%), and Type 4 in 1 case (6%). The proportion of Type 1 in male ankles was 87.5% (7 ankles, n = 8) and 62.5% (5 ankles, n = 8) in female ankles.

Morphometric parameters of the TN branches at Level 1 were measured (Table 1). Two of 7 parameters showed statistically significant difference between male and female specimens (*P* < .05). There was a statistically significant difference between left and right ankles in 2 of 7 parameters (*P* < .05). The median values of the branching points at Level 1 were: −1.8 mm (−14.5 to 10.8 mm), −13.0 mm (−28.8 to −4.5 mm), 7.6 mm (−10.0 to 26.2 mm), and 2.0 mm (−11.2 to 9.0 mm) for LPN, ICN, first MCN branch, and second MCN branch, respectively. The ICN bifurcated more distally in males than in females, with statistical significance (*P* < .05). MCN bifurcated proximal to Level 1 (tip of MM) in 11 of 16 (69%) feet and distal to Level 1 in 5 (31%) feet. The bifurcation point of ICN was distal to Level 1 in 16 (100%) feet. The LPN was found to bifurcate proximal to Level 1 in 4 (25%), distal to in 10 (63%), and at Level 1 in 2 (12%) feet. Of 10 cases in which LPN bifurcated distal to Level 1, 6 (60%) were seen in left, and 4 (40%) were seen in right ankles; the proportion was equal in male and female ankles at 50% each. The median angles between the horizontal line at Level 1 and MPN, LPN, ICN, and the first and second MCN branches were 45°, 60°, 76°, 100°, and 97.5°, respectively (Table 1). The MPN and LPN were found to bifurcate at a greater angle from Level 1 in right ankles when compared to the left (*P* < .05).

**Table 1:** Morphometric parameters of tibial nerve and its branches at the level of the tip of MM.

	Male (n = 8)	Female (n = 8)	*P* value	Left (n = 8)	Right (n = 8)	*P* value	Total (n = 16)
LPN_BP, mm	−3.0 (−14.5 to 10.8)	−1.6 (−8.4 to 9.0)	.6735	−3.0 (−8.4 to 10.8)	−0.8 (−14.5 to 9.0)	.9453	−1.8 (−14.5 to 10.8)
ICN_BP, mm	−19.7 (−28.8 to −11.6)	−11.5 (−22.6 to −4.5)	**.0206**	−12.8 (−28.8 to −6.5)	−13.0 (−21.0 to −4.5)	.2334	−13.0 (−28.8 to −4.5)
MCN1_BP, mm	18.6 (−10.0 to 26.2)	4.3 (−8.4 to 11.6)	.3823	11.3 (−8.4 to 26.2)	3.3 (−10.0 to 21.2)	.1410	7.6 (−10.0 to 26.2)
Angle_MPN, °	49 (45–60)	44.5 (35–50)	**.0247**	45 (35–50)	49 (44–60)	**.0115**	45 (35–60)
Angle_LPN, °	61 (55–75)	56.5 (45–93)	.1507	55 (45–62)	70 (58–93)	**.0169**	60 (45–93)
Angle_ICN, °	77 (72–88)	75.5 (65–95)	1.0000	75.5 (65–88)	79 (72–95)	.4974	76 (65–95)
Angle_MCN1, °	95 (80–112)	101 (90–120)	.5971	94.5 (90–108)	103.5 (80–120)	.4002	100 (80–120)

*P* < 0.05.

Angle_ICN = angle between the ICN and the horizontal line at its branching point, Angle_LPN = angle between the LPN and the horizontal line at the tip of MM, Angle_MCN1 = angle between the first MCN and the horizontal line at the tip of MM, Angle_MPN = angle between the medial plantar nerve and the horizontal line at the tip of MM, ICN = inferior calcaneal nerve, ICN_BP = the branching point of the ICN from the horizontal line at the tip of MM, LPN = lateral plantar nerve, LPN_BP = the branching point of the LPN from the horizontal line at the tip of MM, MCN = medial calcaneus nerve, MCN1_BP = the branching point of the first MCN from the horizontal line at the tip of MM, MM = medial malleolus.

Measurements at Level 2 represented the location of each TN branch within the TT; 3 of 7 parameters exhibited statistical significance between male and female ankles (*P* < .05). The median width of the TT, MM_C, or the distance between the tip of the MM and the midpoint of calcaneus, was 47.9 mm (37.2–57.5 mm); it was 51.6 mm (46.2–57.5 mm) in males and 44.2 mm (37.2–51.2 mm) in females with statistically significant difference (*P* > .0038). The median distances between the tip of the MM and MPN, LPN, and ICN at Level 2 were 21.3 mm (11.2–29.0 mm), 26.9 mm (19.2–38.0 mm), and 29.3 mm (22.0–39.0 mm), respectively. The width between the oblique line at Level 2 and MPN, LPN, and ICN were 5.0 mm (2.8–5.8 mm), 3.4 mm (2.0–5.0 mm), and 1.8 mm (1.0–2.6 mm), respectively (Table 2). Measurements of the TN branches between the tip of MM and the midpoint of calcaneus did not exhibit significant difference between left and right ankles. Furthermore, the median distance between the tip of MM and the MPN at Level 2 were 21.5, 21.9, 11.2, and 17.5 mm for Type 1, 2, 3, and 4, respectively. Bifurcation of the TN branches above the level of the TT was not seen.

**Table 2:** Morphometric data of branches of tibial nerve between the tip of MM and the midpoint of calcaneus.

	Male (n = 8)	Female (n = 8)	*P* value	Left (n = 8)	Right (n = 8)	*P* value	Total (n = 16)
MM_C, mm	51.6 (46.2–57.5)	44.2 (37.2–51.2)	**.0038**	45.6 (38.2–54.3)	49.2 (37.2–57.5)	.3105	47.9 (37.2–57.5)
MPN_MM, mm	24.5 (11.2–29.0)	21.0 (17.5–24.8)	.4295	23.0 (17.5–28.6)	20.0 (11.2–29.0)	.3621	21.3 (11.2–29.0)
LPN_ MM, mm	28.9 (19.2–38.0)	25.5 (21.5–29.0)	.1559	27.3 (23.0–38.0)	26.6 (19.2–33.0)	.3264	26.9 (19.2–38.0)
ICN_ MM, mm	32.1 (22.0–39.0)	28.3 (26.6–32.0)	**.0206**	30.5 (25.4–39.0)	28.9 (22.0–36.0)	.3828	29.3 (22.0–39.0)
MPN_width, mm	5.2 (3.9–6.8)	4.0 (2.8–5.3)	**.0397**	4.7 (3.0–6.8)	5.0 (2.8–6.0)	.6095	5.0 (2.8–6.8)
LPN_width, mm	3.7 (2.7–5.0)	3.1 (2.0–4.0)	.0649	3.4 (2.7–4.2)	3.5 (2.0–5.0)	.5745	3.4 (2.0–5.0)
ICN_width, mm	1.8 (1.0–2.6)	1.7 (1.2–2.5)	.8736	1.9 (1.0–2.6)	1.7 (1.2–2.5)	.7256	1.8 (1.0–2.6)

*P* < 0.05.

ICN = inferior calcaneal nerve, ICN_MM = the distance between the tip of MM and the ICN, ICN_width = the width of ICN between the tip of MM and the midpoint of calcaneus, LPM_MM = the distance between the tip of MM and the LPN, LPN = lateral plantar nerve, LPN_width = the width of LPN between the tip of MM and the midpoint of calcaneus, MM = medial malleolus, MM_C = the distance between the tip of MM and the midpoint of calcaneus, MPN = medial plantar nerve, MPN_MM = the distance between the tip of MM and the MPN, MPN_width = the width of MPN between the tip of MM and the midpoint of calcaneus.

## 4. Discussion

Horwitz^[10]^ first reported the branching pattern of the TN as a classical trifurcation into MPN, LPN, and MCN in the TT. Studies have since explored the variations in the origins of the TN branches in relation to the MM or the malleolar-calcaneal axis (MCA) in order to understand the surgical anatomy of the TTS,^[9,11–14]^ which was first described by Kopell and Thompson,^[15]^ and later established by Lam^[16]^ and Keck.^[17]^ Management of TTS often involves surgical nerve decompression and local nerve block. However, surgery carries risk of injury to the nearby neurovascular bundle, and the resulting scar tissue may lead to recurrence of TTS.^[18]^ Anatomical studies can provide a better understanding of the branching pattern of the peripheral TN branches, which is crucial for performing safe surgical procedures and optimizing patient outcomes.

The terminal branches of the TN supply sensory innervation to the foot. The MPN is responsible for cutaneous supply to the medial two-thirds of the plantar foot, the LPN provides sensation to the lateral one-third of the plantar foot, the MCN supplies the heel pad, and the ICN provides sensory innervation to the anterior aspect of the calcaneus.^[2,19]^ Therefore, the tibial branching pattern and anatomical variations within the TT are clinically significant, and can help guide treatment and diagnosis of TTS. Furthermore, surgical decompression of the TT is much debated to this date, due to the differences in classical bifurcation or trifurcation descriptions of the TN found in textbooks compared to the findings in cadaveric studies.^[5–8]^ This is especially true for the MCN; considerable variations in the number of branches as well as the bifurcation point have been reported.^[8–14]^ Zhang et al^[20]^ found that MCN can have 1 to 3 branches, whereas Yang et al^[18]^ found up to 5 MCN branches. In the present study, 2 MCN branches were seen to originate at the same point (Type 4), which was also described by Yang et al^[18]^ This variation in the number of MCN branches and its origin could be attributed to racial or individual differences.^[18]^

Priya et al^[19]^ demonstrated that the MCN most commonly originates from the LPN, whereas Dellon and Mackinnon,^[11]^ Davis and Schon,^[21]^ and Havel et al^[8]^ all reported that the MCN arises from the MPN. This differed from the current study, which showed that the most common place of bifurcation of the MCN was the TN, and is supported by various studies.^[9,12,21–23]^

Variations in the TN branching pattern and location were seen, in concordance with previous studies. A wide range of classification systems have been reported; Louisia and Masquelet^[23]^ described 2 branching patterns at the level of MCA, Havel et al^[8]^ observed 9 patterns, and Dellon et al^[24]^ found 21 modes of bifurcation patterns. At the level of the TT, Torres and Ferreira^[12]^ found that TN bifurcation occurred distal to the TT in 88% and proximally in 12% of samples. The most common source of medial calcaneal branches was the TN (90%), followed by TN + LPN (4%), and LPN (2%).^[12]^ The most frequently seen number of MCN branches was 1, in 58% of samples, which corresponds to the present study. Although most studies have observed the most common MCN bifurcations to be the TN trunk, followed by mixed presentation (TN, LPN), and LPN, MCN originating from the MPN has also been described by Davis and Schon,^[21]^ Havel et al,^[8]^ and Govsa et al,^[13]^ although not seen in the current study.

The bifurcation distance to the MM or MCA is clinically significant as the MCN’s respective location to the TT may determine risk of symptomatic nerve entrapment if they lie in close proximity to it. In the present study, MCN was found to bifurcate 7.6 mm (−10.0 to 26.2 mm) proximal to MM, and 69% of ankles had MCN bifurcating proximal to the level of the tip of the MM. There was no statistical significant difference between the sexes; the MCN was seen to bifurcate 18.6 mm (−10.0 to 26.2 mm) proximal to MM in males and 4.3 mm (−8.4 to 11.6 mm) proximal to MM in females (*P* < .3823). Ultrasonographic data by Deniel et al,^[25]^ which demonstrated that the distance between the MCN branching point and the MM tip was 7 mm, supports this study’s finding. Oh and Meyer^[2]^ reported that in 35% to 40% of samples, the MCN was found to bifurcate proximally to the TT. Bilge et al^[26]^ found that the distance between the tip of MM and MPN was 26.95 mm; this distance was 21.3 mm (11.2–29.0 mm) in the present study. The distance was similar when comparing male and female, and left and right ankles, at 24.5 mm (11.2–29.0 mm), 21.0 mm (17.5–24.8 mm), 23.0 mm (17.5–28.6 mm), and 20.0 mm (11.2–29.0 mm), respectively, without significant difference in the 2 sets of groups (*P* < .4295, *P* < .3621). These findings are important in identifying the different presentations of TTS; the MCN branches with proximal origins may not penetrate the tunnel, and thus the area of their sensory innervation may remain intact, and can be thought to explain the discrepancy between clinical findings and electrophysiologic tests.

Although TTS is rare, compression of different branches of the TN can present in different ways. For example, patients with MCN compression can present with heel pain radiating to the medial arch of the foot and palpable tenderness over the MM.^[18]^ ICN, also known as Baxter nerve, lies in close proximity to the TT, and is at an increased risk of compression. The resulting Baxter neuropathy presents as heel pain radiating to the medial aspects of the calcaneus and arch of the foot, and is diagnosed by a positive Tinel sign.^[19,27]^ Therefore, detailed descriptions of TN bifurcation patterns, as well as the MCN and ICN origins, which are less reported in current literature, can help guide more precise symptom control. In a study by Deniel et al,^[25]^ ultrasonographic findings of the anatomy of the MCN were within the range of those obtained from cadaveric study. Ultrasound-guided interventions, such as TT release and diagnostic blocks, can result in fewer complications and improved patient outcomes.^[25]^

The current study has several limitations. First, the number of dissected cadavers was small although fresh cadavers were used. Second, this study had methodological constraints such as potential disruption or alteration of anatomical structures because of relying solely on dissection for data collection.

## 5. Conclusion

This morphometric analysis of the TN provides considerable information and detail on the bifurcation patterns of the TN. Although its main limitation lies in the small number of cadavers used, it is the first to provide this level of detail on the anatomical findings of TN branches in a Korean population, to the best of the authors’ knowledge. The goal is to improve the anatomical understanding of the TN branches, with particular emphasis to the MCN and ICN at the level of the TT, to guide a more accurate surgical release of the affected peripheral nerve whilst avoiding inadvertent injuries to smaller nerve branches during surgical procedures in patients presenting with TTS. It may also aid ultrasound-guided intervention for a more effective nerve block in chronic heel pain. With a wide range of classification methods of TN bifurcations being used, there is a need to standardize them. Further studies to look at origins of ICN would be beneficial, as it is poorly documented.

## Author contributions

**Conceptualization:** Im Joo Rhyu, Dong Hwee Kim.

**Data curation:** Jeha Kwon, Soonwook Kwon, Dong Hwee Kim.

**Methodology:** Jeha Kwon, Hong Bum Park, Soonwook Kwon.

**Supervision:** Hong Bum Park, Soonwook Kwon, Im Joo Rhyu, Dong Hwee Kim.

**Writing—original draft:** Jeha Kwon.

**Writing—review & editing:** Im Joo Rhyu, Dong Hwee Kim.
